# A palaeothermometer of ancient Indigenous fisheries reveals increases in mean temperature of the catch over five millennia

**DOI:** 10.1007/s10641-022-01243-7

**Published:** 2022-05-19

**Authors:** Dylan Hillis, Robert Gustas, Daniel Pauly, William W. L. Cheung, Anne K. Salomon, Iain McKechnie

**Affiliations:** 1 Historical Ecology and Coastal Archaeology Laboratory, Department of Anthropology, University of Victoria, 3800 Finnerty Rd, Victoria, BC V8P 5C2 Canada; 2 Institute for the Oceans and Fisheries, University of British Columbia, Vancouver, BC V6T 1Z4 Canada; 3 School of Resource and Environmental Management, Simon Fraser University, Burnaby, BC V5A 1S6 Canada; 4 Bamfield Marine Sciences Centre, Bamfield, BC V0R 1B0 Canada; 5 Hakai Institute, Quadra Island, BC V0P 1H0 Canada

**Keywords:** Archaeology, Climatic change, Indigenous fisheries, Marine historical ecology, Northeast Pacific, Salmon, Zooarchaeology

## Abstract

Climate change is altering the distribution and composition of marine fish populations globally, which presents substantial risks to the social and economic well-being of humanity. While deriving long-term climatic baselines is an essential step for detecting and attributing the magnitude of climate change and its impacts, these baselines tend to be limited to historical datasets and palaeoecological sediment records. Here, we develop a method for estimating the ‘ancient Mean Temperature of the Catch’ (aMTC) using Indigenous fisheries catch records from two archaeological sites in the northeast Pacific. Despite different catch compositions, we observe an increase in aMTC over a 5,000-year period at two contemporaneously occupied archaeological sites in southwestern British Columbia, Canada. We document cooler catches from 5,000 to 3,000 cal yr BP and comparatively warmer catches from 1,800 to 250 cal yr BP. These warmer temperatures are broadly consistent with palaeoceanographic sea surface temperature proxies from British Columbia and Alaska. Because this method requires converting measures of fish bones into estimates of fish size structure, abundance, biomass, and finally aMTC, opportunities exist to account for both variation and uncertainty at every step. Nevertheless, given that preindustrial fisheries data are ubiquitous in coastal archaeological sites, this method has the potential to be applied globally to broaden the temporal and geographic scale of ocean temperature baselines.

**Supplementary Information:**

The online version contains supplementary material available at 10.1007/s10641-022-01243-7.

## Introduction

Warming of the oceans is affecting the distribution, abundance, and productivity of fish populations globally (Cheung et al. 2013; Duarte et al. 2020; Pinsky et al. 2013) and is projected to severely erode the social and economic well-being of coastal communities (Golden et al. 2016; Weatherdon et al. 2016; Wilson et al. 2020). In the northeast Pacific, marine fisheries have supported Indigenous communities for millennia (Brown et al. 2009; McKechnie and Moss 2016; Menzies 2006), a reality that is recognized in Indigenous peoples’ constitutional right to fish for food, social and ceremonial purposes (R.S.C. 1985). As an economic mainstay and source of nutrition and cultural identity, fisheries have existed as a fundamental component of Indigenous communities in British Columbia (BC) throughout the Holocene (Fedje et al. 2005; Moss and Cannon 2011). Indigenous oral histories, ethnographic accounts, and archaeological evidence document the sustained use of a wide range of coastal resources throughout the region where assemblages are often numerically dominated by marine fish bones and shellfish remains (Moss 2012). By an overwhelming margin, fish account for the greatest proportion of vertebrate remains (i.e. identified bone fragments), and Pacific herring (*Clupea pallasii*) and salmon (*Oncorhynchus* spp.) tend to be the most abundant and ubiquitous (McKechnie and Moss 2016; Moss and Cannon 2011). While this evidence indicates an enduring and important role for fisheries in the northeast Pacific, there is no consensus on how zooarchaeological ‘bone counts’ can be translated into estimates of fish abundance, size structure, or biomass.

Currently, most fisheries management decisions rely on data spanning the past several decades to inform present and future population dynamics (Kittinger et al. 2015; McClenachan et al. 2012). Such an approach to fisheries management is fundamentally limited, as modern data lack a deep-time perspective on the history of human fisheries (Steneck and Pauly 2019). Zooarchaeological fisheries data have the potential to provide millennial-scale time series that can reveal long-term variability in oceanographic conditions and ancient catch portfolios. Here, we develop a method based on zooarchaeological analyses of fine-screened fish remains from two Indigenous archaeological sites in coastal BC to estimate relative proportions of fish biomass and ultimately past ocean temperatures, over the past five millennia.

In this paper, we apply the ‘Mean Temperature of the Catch’ (MTC) concept, developed to analyze responses of contemporary fisheries catches to changes in ocean temperature (Cheung et al. 2013), to estimates of fish biomass to back-cast long-term trajectories in past ocean temperatures in the northeast Pacific. Our data indicate that coastal Indigenous fisheries catches reflect cooler ocean temperatures between ca. 5,000 and 3,000 years ago compared to between ca. 1,800 and 250 years ago, both of which are cooler than modern bottom trawl catches. Despite differences in the composition of fish catches between archaeological sites, we observe consistent patterns of increasing MTC. These findings parallel observations from palaeoecological sediment records, indicating long-term ocean warming since the mid-Holocene. Given this is the first estimate of this kind, we highlight the analytical steps and sources of uncertainty in generating temperature estimates, in addition to the opportunities and challenges in applying the Palaeothermometer approach to other archaeological settings and time periods. As coastal archaeological sites with zooarchaeological fisheries data are globally distributed, the method advanced here has the potential to be applied elsewhere. We argue that the Palaeothermometer approach can provide a deep-time perspective on oceanographic variability, the composition of ancient fish catches, and magnitudes of change in the abundance and distribution of fish populations.

## Materials and methods

Fisheries researchers have established that fish species exhibit and maintain through time a preferred temperature range and have subsequently developed methods for detecting shifts in the composition of fisheries as they relate to ocean climate trajectories (Cheung et al. 2013; Pinsky et al. 2013). Cheung et al. (2013) have developed a method for estimating the ‘Mean Temperature of the Catch’ (MTC) and applied it globally to reveal mid-latitude warming trends over a 36-year period. A benefit of the MTC metric is that it can be applied to systematically collected catch data. MTC is one of several climate proxies that are useful for informing coastal communities of the ocean climate challenges that threaten local food security, such as reductions in the catch potential of valuable food fish (Golden et al. 2016; Weatherdon et al. 2016).

### Sample description

**Fig. 1:**
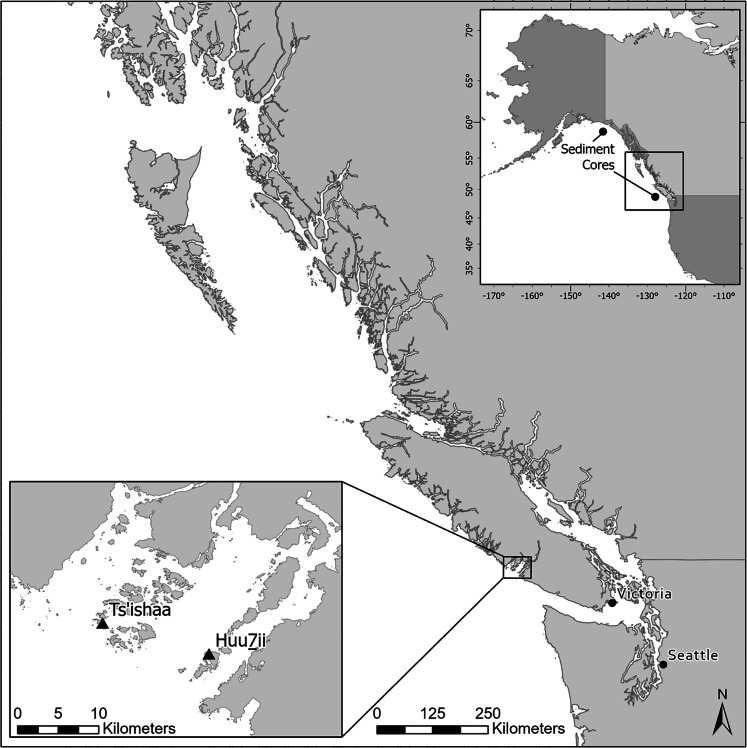
Archaeological fisheries data were collected from two settlement sites in Barkley Sound, on western Vancouver Island, British Columbia, Canada. Ts’ishaa is situated in Tseshaht First Nation Territory, while Huu7ii is located in Huu-ay-aht First Nation Territory. Upper right inset map shows general locations of sediment cores from Praetorius et al. (2015). Map: Robert Gustas

**Fig. 2:**
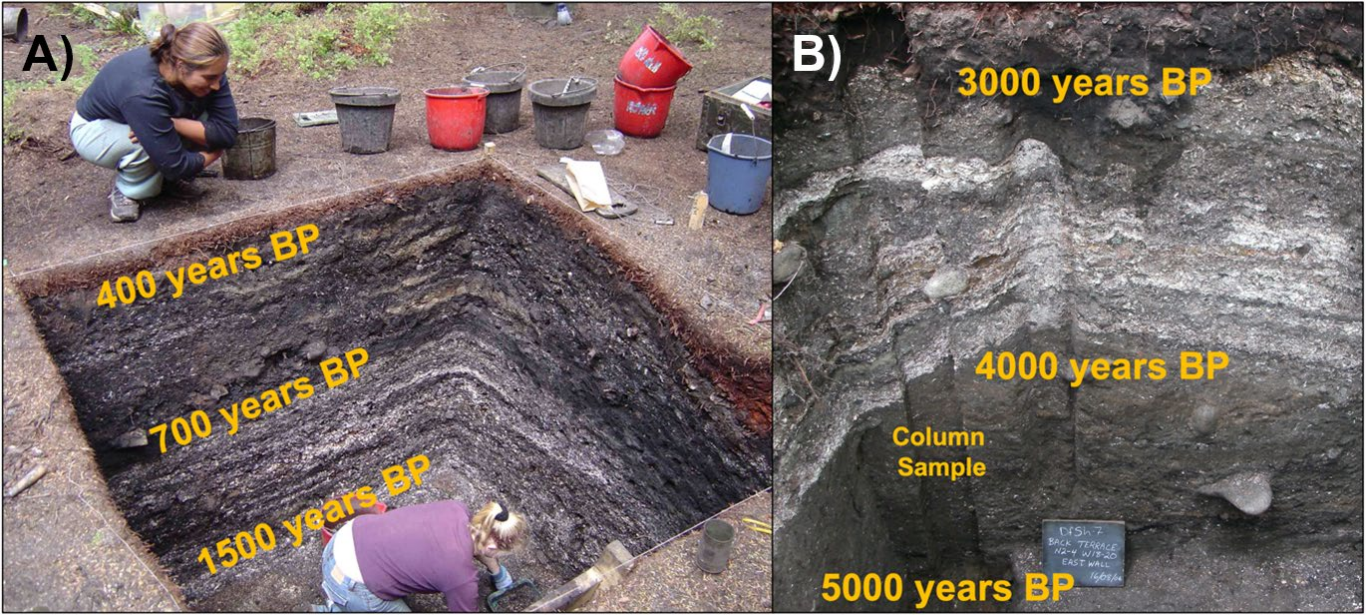
Excavation units and column samples from the archaeological site of Huu7ii, located in Huu-ay-aht First Nation Territory. Panel ***A*** depicts the main village component at Huu7ii, which dates between approximately 1,500–400 yr BP, while panel ***B*** depicts the back terrace component with dates spanning between 5,000–3,000 yr BP. Photos: Iain McKechnie

To estimate changes in ocean temperature using archaeological data and the MTC metric, we first estimated proportional fish biomass from ancient Indigenous catch records. We used zooarchaeological data recovered from fine screen (3.2 and 2 mm) column samples taken from two Indigenous archaeological sites (Ts’ishaa (DfSi-16) and Huu7ii (DfSh-7)) on southwestern Vancouver Island, British Columbia, Canada (Fig. 1). Both archaeological sites are located on small islands (< 2 km^2^) and have contemporaneous occupation histories spanning the past 5,000 years (McMillan and St. Claire 2005, 2012). Enduring human use and occupation of these two village sites is evident in extensive shell-bearing cultural sediments (i.e. shell midden) that can be broadly separated into mid-Holocene (5,000–3,000 yr BP) and late-Holocene (1,800–250 yr BP) components (Fig. 2).

We conducted morphological identifications of recovered skeletal elements to the most specific taxonomic classification possible, using a comparative collection at the University of Victoria, Victoria, BC, Canada (McKechnie 2005b, 2012). The University of Victoria Zooarchaeology Lab holds one of the most extensive comparative collections for Northwest Coast fauna in western North America and has helped inform osteological identifications since 1981 (McKenzie 2021). As not all skeletal elements are morphologically diagnostic to species, some specimens have lower taxonomic resolution required us to create larger taxonomic groupings (Table 1). For osteologically distinct species (e.g. halibut (*Hippoglossus stenolepis*) and lingcod (*Ophiodon elongatus*)), we separated these taxa into their own individual groups rather than the larger taxonomic groupings of flatfish (Order Pleuronectiformes) and greenlings (Family Hexagrammidae).

**Table 1:** Categories of fish taxa used to derive biomass and aMTC estimates, including the genera and species comprising each category; the weighted body mass (kg) for the 25th (Low), 50th (Mid), and 75th (High) empirical quartiles; the weighted median temperature preference (°C; from www.fishbase.org); the sample size (*n*); and the references for body mass estimates

Taxonomic Category^1^	Genus and/or Species	Low (kg)	Mid (kg)	High (kg)	Temp. Pref. (°C)	*n*	References
Anchovy	*Engraulis mordax*^**D**^	0.019	0.021	0.023	11.3	6	McKenzie (2021)
Clingfish	*Gobiesox maeandricus*^**D**^	0.01	0.011	0.014	13.1	4	McKenzie (2021)
Dogfish	*Squalus suckleyi*^**I**^	0.912	1.25	1.778	11.2	3,331	Anderson et al. (2019)
Flatfish	*Eopsetta jordani*^**I**^*, Lepidopsetta bilineata*^**I**^*, Microstomus pacificus*^**I**^*,* Platichthys stellatus*^D^	0.356	0.569	0.867	4.7	17,025	Anderson et al. (2019); DFO (2001)*
Gadids (excluding hake)	*Gadus macrocephalus*^**I**^*, Microgadus proximus*^**I**^	0.182	0.345	0.638	5.5	7,217	Anderson et al. (2019)
Greenling	*Hexagrammos decagrammus*^**D**^*,* *H. stelleri*^**D**^	0.169	0.303	0.529	4.5	83	McKechnie (2007a); McKechnie (2007b)
Gunnel	*Apodichthys flavidus*^**D**^*, Pholis laeta*^**D**^	0.007	0.011	0.024	9.8	12	McKenzie (2021)
Hake	*Merluccius productus*^**I**^	0.094	0.63	0.869	9.8	2,349	Anderson et al. (2019)
Halibut	*Hippoglossus stenolepis*^**D**^	4.399	7.567	13.362	3.0	1,016	Salmen-Hartley (2018)
Herring	*Clupea pallasii*^**D**^	0.088	0.112	0.143	5.0	75,259	DFO (2015); Sanchez (2020)
Lingcod	*Ophiodon elongatus*^**I**^	1.085	2.408	3.689	6.6	2,993	Anderson et al. (2019)
Mackerel	*Pleurogrammus monopterygius*^**D**^	0.455	0.76	0.915	3.4	89	McKenzie (2021)
Midshipman	*Porichthys notatus*^**D**^	0.037	0.064	0.07	11.9	5	McKenzie (2021)
Perch	*Cymatogaster aggregate*^**D**^*, Rhacochilus vacca*^**D**^	0.395	0.475	0.700	11.8	40	McKenzie (2021); Vigneron (2021)
Prickleback	*Anoplarchus purpurescens*^**D**^*, Lumpenus sagitta*^**D**^*, Xiphister atropurpureus*^**D**^*, Xiphister mucosus*^**D**^	0.008	0.01	0.014	8.6	27	McKenzie (2021)
Ratfish	*Hydrolagus colliei*^**I**^	0.27	0.455	0.558	7.4	1,673	Anderson et al. (2019)
Rockfish^2^ (*Sebastes* spp.)	*S. melanops*^**D**^*,* *S. mystinus*^**D**^*,* *S. pinniger*^**D**^*,* *S. nebulosus*^**D**^*,* *S. caurinus*^**D**^*,* *S. maliger*^**D**^*,* *S. proriger*^**D**^*,* *S. brevispinis*^**D**^*,* *S. flavidus*^**D**^*,* *S. nigrocinctus*^**D**^*,* *S. entomelas*^**D**^*,* *S. ruberrimus*^**D**^	0.243	0.556	0.911	6.9	129	McKechnie (2014); McKechnie (2007a); McKechnie (2007b); DFO (2001)
Sablefish	*Anoplopoma fimbria*^**D**^	0.358	0.403	0.546	3.5	36	Nims and Butler (2019)
Salmon^3^ (*Oncorhynchus*)	Weighted Salmon: *O. gorbuscha*^**D**^*,* *O. keta*^**D**^*,* *O. kisutch*^**D**^*,* *O. nerka*^**D**^*,* *O. tshawytscha*^**D**^	3.523	4.308	5.399	4.6	16,045,020	NPAFC (2021)
	Large Salmon: *O. keta*^**D**^*,* *O. tshawytscha*^**D**^	4.26	4.71	5.878	4.5	9,628,390	NPAFC (2021)
	Medium Salmon: *O. gorbuscha*^**D**^*,* *O. keta*^**D**^*,* *O. kisutch*^**D**^*,* *O. nerka*^**D**^*,* *O. tshawytscha*^**D**^	2.18	3.584	4.533	4.7	16,045,020	NPAFC (2021)
	Small Salmon: *O. gorbuscha*^**D**^*,* *O. kisutch*^**D**^*,*	1.716	2.367	3.575	4.9	944,750	NPAFC (2021)
Sand Lance	*Ammodytes hexapterus*^**D**^	0.001	0.002	0.002	4.7	9	McKenzie (2021)
Sculpin^4^	*Enophrys bison*^**D**^, Hemilepidotus hemilepidotus*^D^, Scorpaenichthys marmoratus**^D^	1.297	1.322	1.603	6.2	24	McKenzie (2021)
Skate	*Bathyraja interrupta*^**I**^*, Beringraja binoculata*^**I**^*, Beringraja rhina*^**I**^	0.909	2.244	4.971	5.7	571	Anderson et al. (2019)
Smelt	*Hypomesus pretiosus*^**D**^*, Mallotus villosus*^**D**^, Thaleichthys pacificus*	0.028	0.037	0.037	4.6	125	McKenzie (2021); Anderson et al. (2019)*
Wolf eel	*Anarrhichthys ocellatus*^**I**^	1.84	3.115	4.39	5.9	2	Anderson et al. (2019)

^1^Taxonomic categories are weighted based on the proportion of individual archaeological specimens identified to species level (% MNI) for the Barkley Sound study region (McKechnie and Moss 2016). When such data was not available, we calculated proportionality based on aDNA analysis of archaeological specimens (e.g., Rodrigues et al. 2018), vertebral measurements of archaeological specimens, relative proportions of species caught in the fisheries independent bottom trawl surveys on western Vancouver Island (e.g., Anderson et al. 2019), and locally obtained species in the University of Victoria’s Zooarchaeology Comparative Collection (McKenzie 2021)

^2^Rockfish species proportionality based on Rodrigues et al. (2018), which was used to generate a weighted median temperature preference for the taxonomic category of rockfish. Body mass estimates were obtained using length-to-weight conversion factors for measurements of archaeological specimens (McKechnie 2007a, 2007b; Orchard 2003)

^3^Salmon species proportionality is based on transverse vertebral diameter measurements of archaeological specimens following (Cannon and Yang 2006, 2011; Huber et al. 2011; Miszaniec 2021; Moss et al. 2014; Orchard 2003; Orchard and Szpak 2011). The body mass of salmon is calculated using the proportionality of small (< 8.0 mm), medium (8.0–10.5 mm) and large (> 10.5 mm) vertebrae where the body mass of each species of salmon is a combination of Pink (*Oncorhynchus gorbuscha*) and Coho (*Oncorhynchus kisutch*) (small), all species (medium) and Chum (*Oncorhynchus keta*) and Chinook (*Oncorhynchus tshawytscha*) (large) based on fisheries dependent catch spanning from 1996 to 2019 (NPAFC 2021)

^4^Sculpin species proportionality is based on the relative proportion of the Minimum Number of Individuals (MNI) identified to species level (% MNI)

NOTE: symbols **D** and **I** represent the type of fisheries data that was used to generate body mass estimates. **D** refers to fisheries dependent data (e.g. commercial, recreational, subsistence landings, and archaeological catches), while **I** refers to fisheries independent data (e.g., systematic scientific survey).

 We identified and quantified faunal specimens from multiple areas of the two sites. Samples were taken from vertically spaced 5–10-cm intervals within ‘columns’ of sediment (i.e. column samples) to minimize the probability that bones from a single individual were counted twice (Fig. 2). Based on the ‘Number of Individual Specimens’ (NISP), a ‘Minimum Number of Individual’ (MNI) fish for each archaeological context was then estimated from the largest number of non-repeatable elements within each column sample level. The excavated volume for each discrete column sample level represents 1.0–6.25 L of archaeological sediment and typically contains 10–200 identifiable bone specimens (see Supplementary Table S1). We aggregated taxonomic categories with more than one species unless there were clear size differences in the specimens (e.g. halibut vs. misc. flatfish). Once a MNI for each column sample level was derived, we totaled minimum counts of fish according to the most specific taxonomic division possible (Table 1) and calculated proportional counts for each taxonomic grouping (following McKechnie 2012). The relative proportion of MNI (% MNI) for each taxonomic grouping reflects the percentage of identified fish for each taxonomic category in the archaeological record at both Ts’ishaa and Huu7ii. For closely related species that are not morphologically distinguishable (e.g., rockfish [family Scorpaenidae] and salmon), we refined body mass estimates using existing aDNA identifications (Rodrigues et al. 2018) and bone measurement data for salmon vertebrae (McKechnie 2007a, 2012) from these same sites to estimate the relative composition of species present in the archaeological assemblages (Table 1).

### Deriving biomass from MNI

To derive body mass estimates for fish present in the Barkley Sound zooarchaeological record, we assembled species and genera-specific length and weight data from multiple sources to estimate body mass. We compiled regionally specific fisheries independent data, fisheries dependent data, and where available, archaeological data to best approximate the size structure of fish targeted by Indigenous fishers. For archaeological specimens, we calculated harvested body mass using regression formulae based on skeletal measurements from contemporary specimens of known length and weight (e.g. McKechnie 2007a; Nims and Butler 2019; Orchard 2003; Salmen-Hartley 2018; Sanchez 2020; Table 1). In the absence of archaeological data, we preferentially used contemporary regionally specific fisheries independent scientific survey data (Anderson et al. 2019), dockside (recreational) survey data from Barkley Sound (DFO 2001), size and weight data for comparative specimens in the University of Victoria’s Zooarchaeology Lab (McKenzie 2021), and finally, more general estimates from archaeological and scientific literature (e.g. NPAFC 2021). For each species, we compiled multiple length and weight estimates and preferentially selected sources using the ranking described above (see Supplementary Table S2).

We then calculated the median body mass (kg) for each taxonomic category by weighting each species contribution based upon the relative proportion of MNI observations (% MNI) for each species present in the archaeological record. For greater detail on the process used to calculate weighted body mass estimates, see Table 2. In addition to using the median body mass for each taxonomic group, we report the 25th and 75th quartiles to enable uncertainty estimates for biomass.

**Table 2:** Barkley Sound sculpin example detailing the process used in this study to derive weighted body mass estimates and weighted temperature preferences for each taxonomic category of fish. In this example, sculpin bones (recovered from both Ts’ishaa and Huu7ii) have been identified to the lowest taxonomic level possible. These data indicate the presence of three distinct species (Buffalo Sculpin (*Enophrys bison*), Cabezon (*Scorpaenichthys marmoratus*), Red Irish Lord (*Hemilepidotus hemilepidotus*)), one genus level designation (Irish Lord spp.), and one broader family level designation (Sculpin spp.). When Minimum Number of Individuals (MNI) is summed, the taxonomic category of ‘Sculpin’ represents a minimum of 49 individual fish. The MNI for each species, genus and family is then divided by the total MNI to derive proportion (%). For broader level designations (e.g. Irish Lord spp. and Sculpin spp.), body mass and temperature preferences are informed by the proportion of species level MNI counts comprising each group. In this case, Irish Lord spp. is comprised entirely of Red Irish Lord, while Sculpin spp. is weighted based on the proportion of Buffalo, Cabezon, Red Irish Lord, as well as Irish Lord spp. In other words, the 10 MNI for Sculpin spp. are comprised of all lower-level identifications based upon their proportions. Once the % contribution of each species, genus and family to the taxonomic category ‘Sculpin’ is known, a weighted body mass and weighted temperature preference can be generated. This is done by multiplying each species’ body mass and temperature preference by their proportion (%) and then summed. The product of this is then divided by the sum of proportions for each species, genus and family level designations

						Taxonomic Category
Species	Buffalo Sculpin	Cabezon	Irish Lord spp.	Red Irish Lord	Sculpin spp.	Sculpin
MNI	2	12	16	9	10	49
Proportion (%)	4.1	24.5	32.7	18.4	20.4	100
Temperature preference (°C)	9.3	9.6	4.3	4.3	6.2	6.2
Median body mass (kg)	0.047	3.629	0.316	0.316	1.322	1.322

For larger taxonomic categories including multiple species (e.g. salmon and sculpin (Family Cottidae)), we calculated body mass by weighting the individual body mass of each species by the proportion of specimens that had been identified by skeletal measurements (Cannon and Yang 2006; Cannon and Yang 2011; Huber et al. 2011; Miszaniec 2021; Moss et al. 2014; Orchard and Szpak 2011; see Table 2). In the case of salmon, we calculated the body mass for each species using body mass data compiled from contemporary fisheries dependent catch landings (NPAFC 2021). We then weighted each salmon species’ contribution to the larger taxonomic group using skeletal measurement data of archaeological specimens (*n* = 282) based on the size distribution of salmon vertebral measurements. Finally, we multiplied the MNI by the body mass for each taxonomic category and column sample to estimate biomass (kg). These total weights were summed and divided by the total biomass for each archaeological assemblage to determine the proportion of the catch (% Biomass). Table 3 provides a step-by-step walk-through of a hypothetical archaeological assemblage for calculating % Biomass from MNI data.

**Table 3:** Generating % Biomass from a hypothetical archaeological assemblage. In this example, the Minimum Number of Individuals (MNI) for five taxonomic categories of fish are used to derive % Biomass. Using the weighted body mass (kg) for each group of fish, body mass is multiplied by MNI to generate biomass (kg). Biomass is then summed across all taxonomic categories to calculate the proportion of the catch for each taxonomic category

Taxonomic category	Anchovy	Halibut	Herring	Perch	Rockfish	Sum of biomass (kg)	Sum of the proportion of the catch (%)
MNI	15	1	30	10	8		
Body mass (kg)	0.021	7.567	0.112	0.478	0.556		
Biomass (kg)	0.315	7.567	3.36	4.78	4.448	20.47	
% Biomass	1.5	37.0	16.4	23.4	21.7		100

### Calculating ancient MTC (aMTC)

The median temperature preference for each species present in the Barkley Sound zooarchaeological record was obtained from www.fishbase.org (Froese and Pauly 2021). We then calculated a weighted median temperature preference for each taxonomic category based on the proportion of species present in the region’s zooarchaeological record (% MNI). The median temperature preference for each taxonomic group was then used to calculate ancient MTC (aMTC) for each archaeological assemblage.

Following Cheung et al. (2013), aMTC was calculated using the equation provided below:

$${aMTC}_{tp}= \frac{{\Sigma }_{i}^{n}{T}_{i}{C}_{i,tp}}{{\Sigma }_{i}^{n}{C}_{i,tp}}$$

Here, ${C}_{i,tp}$ is the catch of taxonomic category *i* in a specific archaeological assemblage for a temporal period *tp*, ${T}_{i}$ is the median temperature preference of taxonomic category *i*, and *n* is the total number of taxonomic categories. In other words, aMTC is computed by multiplying the median temperature preference for each taxonomic category by the proportion of the catch (% Biomass). We then summed these results and multiplied the product by the total count of groups for each archaeological site and temporal period. These results were then divided by the sum of the proportion of the catch (i.e. 100%) multiplied by the number of groups for each assemblage and temporal period to derive aMTC estimates.

### Calculating modern MTC

To compare aMTC to a modern fishery dataset, we similarly examined catch records from the systematic fisheries independent Groundfish Synoptic Bottom Trawl Surveys, conducted biannually between 2004 and 2018 by Fisheries and Oceans Canada off western Vancouver Island (Anderson et al. 2019). We summed the total catch landings (kg) over this 14-year period for each species and then summed the total biomass for this temporal period. Following this, we divided each species’ biomass by the total biomass to calculate the proportion of the catch (% Biomass). These results were then multiplied by the median temperature preference for each species representing more than 1% of the catch (*n* = 40 species). We then summed the product of the median temperature preferences multiplied by the proportion of the catch. Next, these results were multiplied by the total number of species (*n* = 40). Finally, to derive modern MTC, we divided the product of the sum multiplied by the total number of species by the sum of the proportion of the catch multiplied by the number of species.

### Chronology and radiocarbon dating

The two mid-to-late Holocene archaeological assemblages used in this study were dated by 50 radiocarbon samples from stratigraphically associated terrestrial charcoal (see Supplementary Table S3). The two broad age-ranges were further separated by geomorphological context, including mid-Holocene deposits on elevated terraces away from the modern shoreline and lower elevation deposits adjacent to contemporary shorelines. These site formation patterns are consistent with relative sea level histories for the region (Friele and Hutchinson 1993). Calibrated radiocarbon dates for the late-Holocene fish assemblages at Ts’ishaa and Huu7ii date to 1,800–250 yr BP and 1,500–400 yr BP, respectively. The mid-Holocene assemblages for both sites date between approximately 5,000–3,000 calibrated yr BP using the Intcal20 curve (see Supplementary Table S3).

## Results

### MNI estimates

**Fig. 3:**
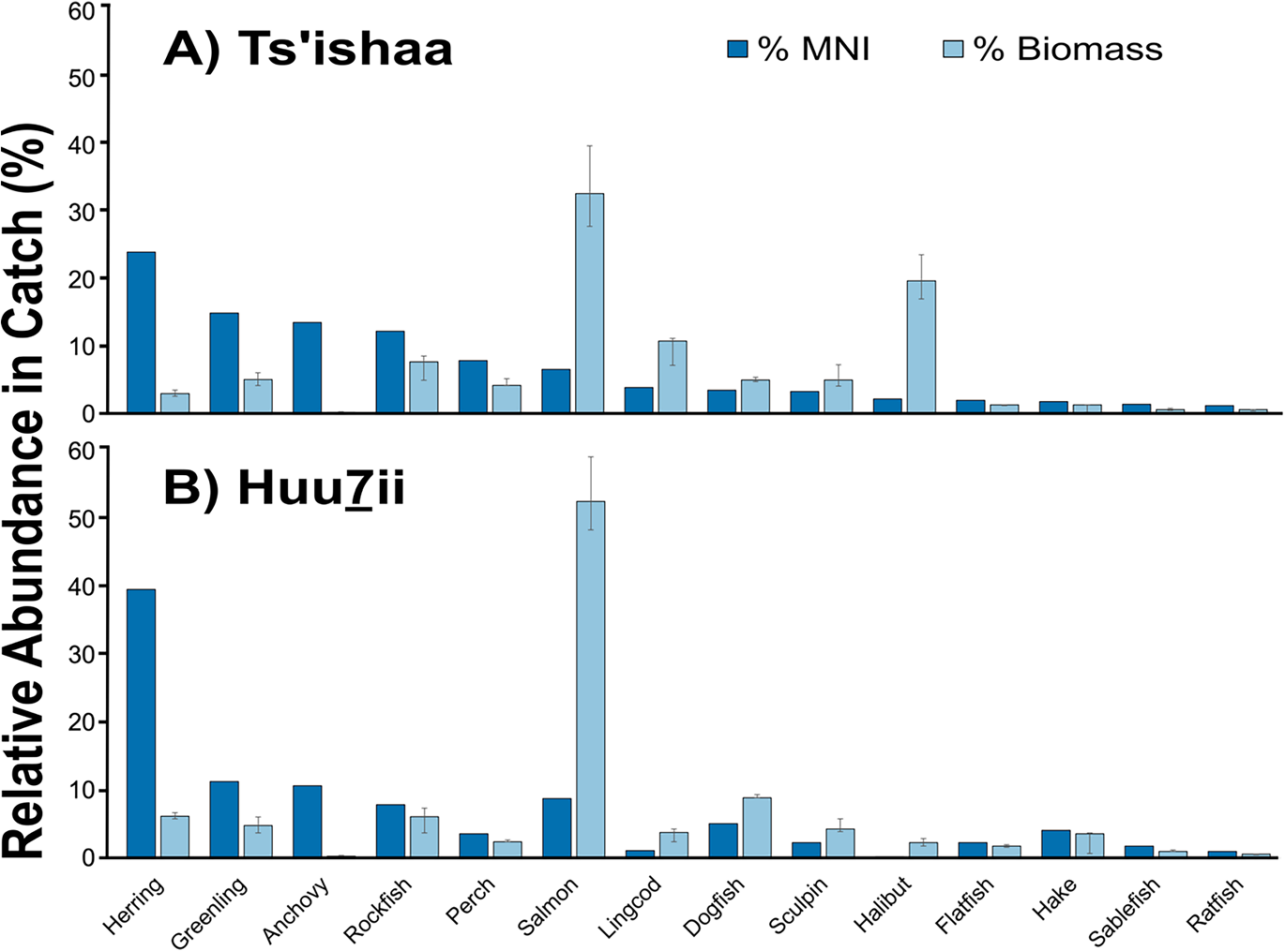
Taxonomic composition of fish at two archaeological sites in British Columbia, Canada, over the past five millennia. Bars depict the relative proportion of the Minimum Number of Individuals (% MNI) recovered from column samples and the relative proportion of biomass (% Biomass) estimated from median body mass values for each taxonomic group (Table 4). Error bars represent % Biomass using the 25th and 75th quartiles for body mass estimates

Throughout both temporal periods, the greatest number of individual specimens (NISP) recovered from Ts’ishaa and Huu7ii are forage fish (e.g. Pacific herring and northern anchovy (*Engraulis mordax*)). When NISP were converted to MNI estimates (% MNI), forage fish were still the most numerous fish represented at both sites (Fig. 3). However, when contrasting NISP and MNI, we found that MNI estimates tended to elevate the importance of salmon. Due to the distinctive genus-specific texture of fragmentary salmon vertebrae which are easier to confidently identify (Cannon and Yang 2011), the frequent presence of small salmon vertebrae fragments may contribute to an overrepresentation in MNI counts. In the context of calculating MNI from small volume fine screened column samples, the presence of individual bone fragments from salmon has a stronger influence on MNI counts than more numerically abundant but smaller taxa (e.g. forage fish). This is because forage fish have smaller skeletal elements which are more challenging to confidently identify than salmon bones when fragmented.

### Biomass estimates

When MNI is subsequently converted to proportional biomass (% Biomass), salmon are estimated to contribute the single greatest fraction to fish biomass (i.e. total weight of fish caught) at both Ts’ishaa and Huu7ii (Fig. 3). At Ts’ishaa, halibut ranked second after salmon, followed by lingcod, rockfish, and greenling. In contrast, proportional biomass at Huu7ii indicates that dogfish (*Squalus suckleyi*) ranked second, followed by herring, rockfish, and greenling. These trends reveal differences in the composition of fish catches across sites despite being in relative proximity (< 15 km). In contrast to MNI, biomass estimates document a substantial reduction in the rank order of forage fish and a dramatic increase in salmon, halibut, and other large-bodied fishes, as would be expected when bone counts are converted to numbers of fish and then biomass.

Temporal trends show modest differences in the rank order of fish in the mid and late-Holocene components at both archaeological sites (Table 4), indicating the persistence of Indigenous fisheries and fish populations over broad time scales. Throughout the mid-Holocene, halibut represented the largest proportion of the catch (28%) at Ts’ishaa followed by salmon, lingcod, greenling, and rockfish. This differs from the late-Holocene assemblage at Ts’ishaa, where salmon (35%) dominated, followed by halibut, lingcod, rockfish, and sculpin. Meanwhile, the mid-Holocene period at Huu7ii indicates a strong role of salmon (53%) followed by herring, dogfish, greenling, and rockfish. The late-Holocene period continues to show salmon as the highest proportion of the catch by weight (53%), followed by dogfish, rockfish, sculpin, and hake (*Merluccius productus*).

**Table 4:** Comparison of the relative proportion of the Minimum Number of Individuals (% MNI) and the relative proportion of the estimated catch (% Biomass) for each archaeological site and temporal period under consideration. % Biomass is calculated using the median body mass estimate multiplied by MNI counts for each taxonomic grouping

	Ts’ishaa % MNI		Ts’ishaa % Biomass		Huu7ii % MNI		Huu7ii % Biomass	
Approx. Age (cal yr BP)	5,000–3,000	1,800–250	5,000–3,000	1,800–250	5,000–3,000	1,500–400	5,000–3,000	1,500–400
Anchovy	7	16	< 1	< 1	7	13	< 1	< 1
Dogfish	3	4	4	6	3	6	9	9
Flatfish	2	2	1	2	< 1	3	< 1	2
Greenling	24	13	8	4	11	12	7	4
Hake	< 1	3	< 1	2	< 1	6	< 1	5
Halibut	4	2	28	17	< 1	< 1	3	2
Herring	27	23	3	3	62	28	15	4
Lingcod	5	4	13	10	< 1	2	1	5
Perch	10	7	5	4	5	3	5	2
Ratfish	1	1	< 1	1	1	1	1	1
Rockfish	11	13	7	8	4	10	5	7
Sablefish	1	2	< 1	1	< 1	3	< 1	1
Salmon	6	7	28	35	6	10	53	53
Sculpin	< 1	4	< 1	7	1	3	2	5

### MTC calculations

**Fig. 4:**
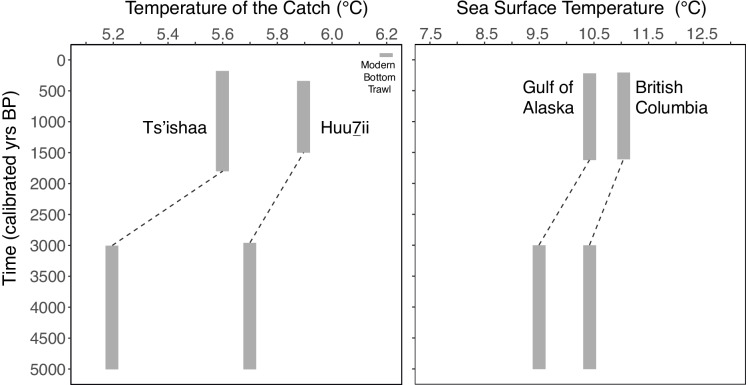
Estimates of ancient Mean Temperature of the Catch (aMTC) presented in this study alongside detrended Sea Surface Temperature (SST) reconstructions from palaeooceanographic sediment cores (Praetorius et al. 2015). Temperature values for aMTC results are derived from the median temperature preference and median body mass estimate for each taxonomic group under study. Dashed lines indicate gaps in the zooarchaeological record (i.e. no data present for this temporal period)

Comparisons of the aMTC from both temporal components at Ts’ishaa and Huu7ii indicate cooler fish catches during the mid-Holocene (5.24℃ and 5.73℃, respectively) followed by warmer temperatures during the late-Holocene occupation period (5.64℃ and 5.9℃, respectively) (Fig. 4). At Ts’ishaa, the range for aMTC using the median body mass estimate during the mid-Holocene is 5.22 to 5.3℃, while the range is 5.26 to 6.01℃ for the late-Holocene component. Meanwhile, the range at Huu7ii during the mid-Holocene is 5.52 to 6.06℃, while the range is 5.46 to 6.15℃ for the late-Holocene occupation period.

When aMTC is calculated using the 25th and 75th empirical quartiles for body mass estimates, the data reveal similar increases in the temperature of fish catches. For instance, the mid-Holocene assemblage at Ts’ishaa using the 25th quartile body mass estimate is 5.3℃ (range of 5.28 to 5.34℃) and when using the 75th quartile body mass estimate, aMTC is 5.15℃ (range of 5.12 to 5.22℃). At the same time, aMTC for Huu7ii using the 25th quartile is 5.7℃ (range of 5.5 to 6.02℃) and for the 75th quartile aMTC is 5.79℃ (range of 5.55 to 6.17℃). During the late-Holocene occupation period at Ts’ishaa, aMTC is 5.61℃ (range of 5.22 to 5.96℃) using the 25th quartile body mass estimate and 5.59℃ (range of 5.19 to 6.02℃) using the 75th quartile. At Huu7ii, the late-Holocene aMTC estimate using the 25th quartile is 5.68℃ (range of 5.34 to 5.87℃) and when using the 75th quartile, aMTC is 5.97℃ (range of 5.52 to 6.2℃). These increases in temperature are supported by geochemical data from marine sediment cores taken along the continental shelf in both BC and in the Gulf of Alaska (Praetorius et al. 2015). When aMTC is calculated for each respective archaeological site (i.e. representing five millennia of fishing effort), aMTC at Ts’ishaa using the median body mass estimate is 5.54℃ (5.53℃ and 5.47℃ for the 25th and 75th quartiles, respectively) while it is 5.86℃ (5.68℃ and 5.93℃ for the 25th and 75th quartiles, respectively) at Huu7ii. Most strikingly, all aMTC estimates are lower than the modern MTC calculated from the western Vancouver Island bottom trawl surveys (6.2℃).

## Discussion and conclusion

These estimates of millennial-scale increases in ocean temperature measured by ancient fisheries catches and supported by geochemical data from marine sediment cores represent the first application of the MTC method to preindustrial fisheries records using archaeological data. Considering that coastal archaeological sites with fisheries records are present across the globe, this research methodology illuminates the potential for detecting shifts in fisheries from myriad locations and timescales. This method involves several assumptions and sources of uncertainty suitable for future refinement.

**Fig. 5:**
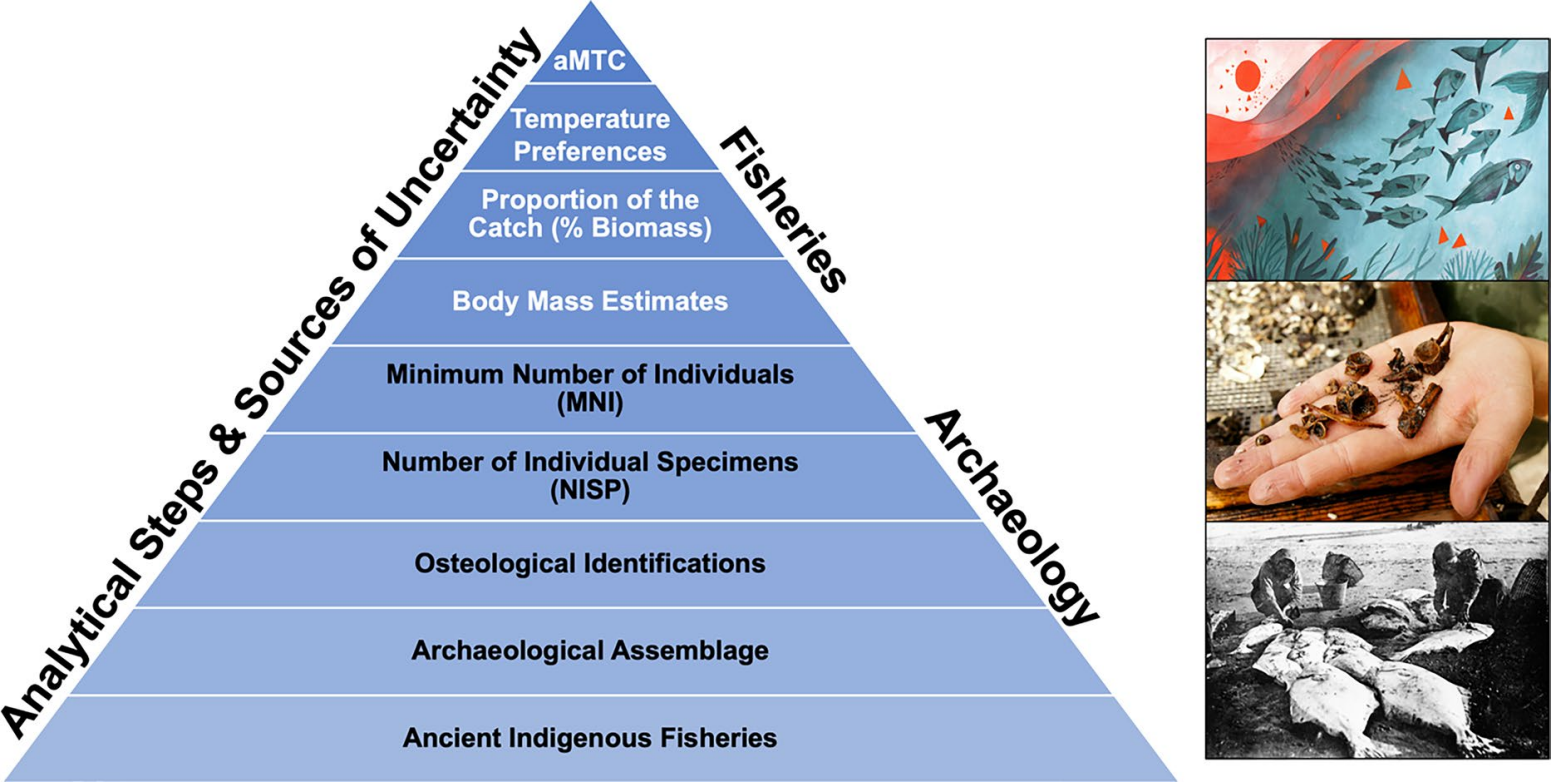
Analytical steps taken to generate aMTC estimates from zooarchaeological fish remains, representing five millennia of human fishing activity in the study area. Each analytical step is associated with its own uncertainty and assumptions. Sources of uncertainty include (from bottom to top of pyramid): (1) ancient Indigenous fisheries are inherently selective records (i.e. 'fisheries dependent' data) that do not provide a complete estimate of marine community structure; (2) archaeological assemblages may not be representative of ancient Indigenous fisheries, due to taphonomic and cultural factors as well as recovery methods (e.g. screen size, sampling effort, and fragmentation); (3) limitations around osteological identifications, which are influenced by the skeletal morphology of different taxa (e.g. robustness of certain skeletal elements, differences in the number of elements between taxa, and identifiability of elements), the skill of each analyst conducting identifications, and the size of the comparative collection; (4) how the Number of Individual Specimens (NISP) is generated from zooarchaeological analyses, which is largely determined by the identifiable portions of skeletal elements; (5) how the Minimum Number of Individuals (MNI) is derived from NISP (i.e. the number of individuals based upon non-repeatable elements) and the effect that study design can have on shaping how different stratigraphic layers are considered distinct from one another; (6) uncertainty around the body mass of ancient Indigenous fish catches and how this effects relative biomass (% Biomass) calculations; (7) the challenge of using temperature preferences of modern fish to approximate the temperature preferences of ancient fish, as evolutionary and/or geographic shifts may have occurred over long time spans; and finally, (8) uncertainty around using aMTC as a temperature proxy of ancient fish catches. Photo credits (top to bottom): Luisa Rivera/Yale E360, Dylan Hillis, unknown (Washington State Archives 1890–1910: AR-07809001-ph003398)

We acknowledge multiple sources of uncertainty in developing the aMTC index from zooarchaeological bone count data (Fig. 5). Many recognize that ancient fishing practices may not be fully represented in the archaeological record. For instance, cultural factors can have a large influence on what gets preserved in an archaeological deposit, including transport, processing techniques for consumption, storage, trade, as well as spiritual considerations. In addition, biogeochemical taphonomic processes shape the formation of archaeological deposits including burial conditions, and differential preservation and fragmentation (Gifford-Gonzalez 2018). Furthermore, archaeological recovery methods such as column sampling using fine mesh sizes dramatically increase the number of elements recovered and despite smaller examined volumes, reveal equivalent measures of species richness with greater accuracy of taxonomic proportionality (McKechnie 2005a).

Another concern is the accuracy and specificity of osteological identifications and the representativeness of comparative collections. In many cases, a substantial percentage of fragmentary bone specimens cannot be identified. These limitations mean that the representativeness of zooarchaeological data as a record of ancient fish landings is incomplete. As is well recognized in zooarchaeology, MNI is fundamentally derived from bone specimen counts and can be calculated across sites levels which affects how many organisms in an archaeological context are counted (e.g., how different layers are distinct from one another). Another challenge of using zooarchaeological data to generate aMTC relates to the applicability of body mass estimates as the size, length, and body mass of fish can differ between ancient and modern fisheries data (Braje et al. 2017; McKechnie 2007a). Thus, drawing upon contemporary data to estimate ancient fish populations has limitations. Finally, median temperature preferences are based on a relationship between environmental conditions and contemporary species occurrence that may not directly reflect physiological temperature preferences, evolutionary shifts in fish physiology, and temperature association given large-scale ecosystem shifts in the past.

To address these limitations, we focused our analysis on two archaeological sites close to each other and with similar occupational histories, analytical methods, and rigorous sampling and quantification techniques. We then compiled an array of body mass estimates and where possible, used fisheries independent data or body mass estimates derived from archaeological assemblages using regressions or body size comparisons.

A substantive consideration relates to the accuracy of MTC as a temperature proxy, as well as the temporal resolution of archaeological data that spans millennial time scales. Such detrended estimates undoubtedly incorporate a range of climatic states that homogenize climatic variability. However, such time-averaged data also reduces potential confounding effects of fishing effort, seasonality in site use, fishing technology, and variability in climate. As the data reported on here reflect Indigenous communities living on small islands and consuming fish on a regular basis, changes in the mode of fishing over time may be independent of changing ocean temperature. To better account for the uncertainty and error in our aMTC estimates, uncertainty could be propagated throughout our calculations with hierarchical Bayesian methods whereby specified prior distributions could be associated with each estimated parameter and its associated uncertainty. Furthermore, Monte Carlo simulation methods (Yanai et al. 2010) could be used to better characterize the variability associated with MNI counts, as well as body mass and temperature preferences, as it would consider the distribution of data rather than a single summary value. Such an approach to estimating the uncertainty and error associated with aMTC estimates would strengthen the Palaeothermometer method, as it would allow for the deployment of various statistical tests.

Over the past five millennia, the composition of the catch and aMTC estimates reflect modest differences between the two villages of Ts’ishaa and Huu7ii. The difference in aMTC between sites is consistent across the two contemporaneous occupation periods, which is indicative of climatic shifts operating at a regional scale throughout the Holocene (Fig. 4). The modest differences in the composition of the catch (% Biomass) between sites is intriguing, as both sites are close to one other (< 15 km), as well as being situated on small islands (< 2 km^2^). We interpret the difference in aMTC to reflect both the local bathymetry and habitat characteristics in proximity to each village, as well as social and cultural histories associated with these politically separate communities. The two villages are associated with different contemporary First Nations and their respective territories (Tseshaht and Huu-ay-aht), which are culturally associated with territorial access to different salmon rivers, spawning grounds, rocky reef habitats, as well as delineated offshore fishing banks (McMillan and St. Claire 2005; 2012). The comparatively warmer aMTC values for Huu7ii may reflect the use of protected nearshore waters with a greater abundance of salmon than at the more exposed site of Ts’ishaa, which has significantly more halibut. It is important to recognize that the inhabitants of these communities would have targeted specific species of fish at varying depths in the water column. This is an important consideration, as the fisheries independent bottom trawl survey data (i.e., Anderson et al. 2019) used for the modern baseline relies upon catch data from greater depths than traditional fishing methods would have allowed. As a consequence, modern fisheries independent survey data may miss important components of a cultural fishery (e.g., salmon). Therefore, depending on the depths local fishers were accessing in the past, aMTC would reflect a homogenization of fishing effort based on the temperature preferences associated with different species of fish and their location within the water column.

As is demonstrated in our results despite the complexities listed above, the two site assemblages of Ts’ishaa and Huu7ii reveal coherent and similar trends of increasing ocean temperature over the past 5,000 years. Furthermore, our data indicate MTC is higher in the historic era, which corroborates reported increases in MTC over the past 36 years (Cheung et al. 2013; Ng and Cheung this issue). Collectively, these results indicate that preindustrial fisheries in the northeast Pacific had cooler temperatures than at present times. These findings are supported by detrended data from marine sediment cores which shows similar increases in two different regions of the northeast Pacific (Praetorius et al. 2015). Additionally, geochemical analysis of sediment cores from southwestern Yukon (Anderson et al. 2007), faunal records preserved in marine sediments in Barkley Sound (Wright et al. 2005), and geochemical analysis of archaeologically recovered fish bones from Barkley Sound (Monks 2017) reflect a similar shift in climate throughout the mid and late-Holocene. By integrating zooarchaeological and geochemical data, we provide multiple lines of evidence that document broad ocean warming in the northeast Pacific over the past five millennia. While marine sediment core records offer a regional-scale perspective of oceanographic variability, the value of our aMTC results are in their ability to show how local community fisheries responded to variability in the distribution and abundance of fish populations over time.

Estimating long-term climatic baselines is an essential step for understanding the magnitude of change in fisheries activities, particularly if used to better inform recovery targets that foster social and ecological well-being. This study contributes to broadening the relevance of zooarchaeological bone count data and biomass estimates in relation to research into global environmental change. The aMTC method can be further refined by quantifying uncertainty and expanded to include other archaeological datasets from different latitudes and cultural settings. Future efforts should include refinement of size estimates for zooarchaeological fish remains, species identifications, as well as quantifying variation and uncertainty at every step. While the method described here has the potential to inform preindustrial fisheries baselines globally, we acknowledge that the successful application of this approach requires detailed site-specific zooarchaeological data to inform biomass estimates. For greater consistency in the application of this method, coastal archaeologists are encouraged to develop region-specific body mass estimates for fish taxa informed by archaeological size reconstructions. In this way, the Palaeothermometer approach can be expanded to other geographic regions and temporal periods to inform preindustrial fisheries baselines.

## Permits and permissions

Archaeological fieldwork research permits were issued by elected council resolutions from Tseshaht and Huu-ay-aht First Nations for respective archaeological excavation during the Tseshaht Archaeological Project (1999–2001) and the Huu-ay-aht Archaeological Project (2004–2006) co-directed by Denis St. Claire and Alan McMillan. Additional permits were obtained from Parks Canada and the British Columbia Archaeological Branch.

## Supplementary Information

Below is the link to the electronic supplementary material.

Supplementary file1 (XLSX 187 KB)

Supplementary file2 (DOCX 49 KB)

## Data Availability

Data posted in the supplementary material at 10.1007/s10641-022-01243-7. Primary zooarchaeological data available by request to IM who will coordinate permission of respective First Nations.

## References

[CR1] Anderson L, Abbott MB, Finney BP, Burns SJ (2007). Late Holocene moisture balance variability in the southwest Yukon Territory, Canada. Quat Sci Rev.

[CR2] Anderson SC, Keppel EA, Edwards AM (2019). A reproducible data synopsis for over 100 species of British Columbia groundfish,&nbsp;Canadian Science Advisory Secretariat (CSAS) Research Document 2019/041.

[CR3] Braje TJ (2017). Historical ecology and the conservation of large, hermaphroditic fishes in Pacific Coast kelp forest ecosystems. Sci Adv.

[CR4] Brown F, Brown YK (2009) Staying the course, staying alive: coastal First Nations fundamental truths: biodiversity, stewardship and sustainability. Biodiversity BC, Victoria

[CR5] R.S.C. Canada (1985) Fisheries Act RSC., F-14, s 1. https://laws.justice.gc.ca/eng/acts/f-14/index.html

[CR6] Cannon A, Yang DY (2006). Early storage and sedentism on the Pacific Northwest coast: ancient DNA analysis of salmon remains from Namu. British Columbia Am Antiq.

[CR7] Cannon A, Yang DY (2011). Pushing limits and finding interpretive balance: a reply to monks and orchard. American Antiquity.

[CR8] Cheung WWL, Watson R, Pauly D (2013). Signature of ocean warming in global fisheries catch. Nature.

[CR9] DFO (2001). West Coast Vancouver Island sport fishery creel survey statistics 2001 and historical data 1984–2000 / by DM Lewis.

[CR10] DFO (2015). West Coast Vancouver Island test Seine samples sections: 231, 232, 233, 1975–2010.

[CR11] Duarte CM (2020). Rebuilding marine life. Nature.

[CR12] Fedje DW, Mackie AP, Wigen RJ, Mackie Q, Lake C, Fedje DW, Mathewes RW (2005). Kilgii Gwaay: an early maritime site in the south of Haida Gwaii. Haida Gwaii: Human history and environment from the Time of Loon to the Time of the Iron People.

[CR13] Friele PA, Hutchinson I (1993). Holocene sea-level change on the central west coast of Vancouver Island, British Columbia. Can J Earth Sci.

[CR14] Froese R, Pauly D (2019) FishBase https://www.fishbase.org/ FishBase Consortium, Quantitative Aquatics, Inc., Los Banos, Philippines

[CR15] Gifford-Gonzalez, D. P. 2018. An Introduction to Zooarchaeology. Springer, New York

[CR16] Golden CD (2016). Nutrition: fall in fish catch threatens human health. Nature.

[CR17] Huber HR, Jorgensen JC, Butler VL, Baker G, Stevens R (2011) Can salmonids (*Oncorhynchus* spp.) be identified to species using vertebral morphometrics? J Archaeol Sci 38(1):136–146

[CR18] Kittinger JN, McClenachan L, Gedan KB, Blight LK (2015) Marine historical ecology in conservation: applying the past to manage for the future. University of California Press, Oakland

[CR19] McClenachan L, Ferretti F, Baum JK (2012) From archives to conservation: why historical data are needed to set baselines for marine animals and ecosystems. Conserv Lett 5(5):349–359. 10.1111/j.1755-263X.2012.00253.x

[CR20] McKechnie I (2005a) Column sampling and the archaeology of small fish at Ts’ishaa. In: McMillan AD and St. Claire DE (eds) Ts'ishaa: Archaeology and Ethnography of a Nuu-chah-nulth Origin Site in Barkley Sound., pp. 206–223. Archaeology Press, Simon Fraser University, Burnaby

[CR21] McKechnie I (2005b) Five thousand years of fishing at a shell midden in the Broken Group Islands, Barkley Sound, British Columbia. MA Thesis, Department of Archaeology, Simon Fraser University, Burnaby

[CR22] McKechnie I (2007a) Investigating the complexities of sustainable fishing at a prehistoric village on western Vancouver Island, British Columbia. Canada J Nat Conserv 15(3):208–222

[CR23] McKechnie I (2007b) Vertebrate faunal analysis at Himayis (205T, DeSi-17) and the Clarke Island Defensive Site (212T, DeSi-26), Broken Group Islands, Pacific Rim National Park Reserve. Parks Canada, Cultural Resource Services, Victoria 1–68

[CR24] McKechnie I (2012) Zooarchaeological analysis of the Indigenous fishery at the Huu7ii Big House and Back Terrace, Huu-ay-aht Territory, Southwestern Vancouver Island. In: McMillan AD and St. Claire DE (eds) Huu7ii: Household Archaeology at a Nuu-chah-nulth Village Site in Barkley Sound, Archaeology Press, Simon Fraser University, Burnaby 154–186

[CR25] McKechnie I (2014) An archaeology of food and settlement on the Northwest Coast. PhD Dissertation, Department of Anthropology, University of British Columbia, Vancouver

[CR26] McKechnie I, Moss ML (2016). Meta-analysis in zooarchaeology expands perspectives on Indigenous fisheries of the Northwest Coast of North America. J Archaeol Sci.

[CR27] McKenzie K (2021) University of Victoria - Zooarchaeology Lab Collection v1.1. University of Victoria, Victoria, 10.5886/jej09d

[CR28] McMillan AD, St. Claire DE (2005) Ts’ishaa: archaeology and ethnography of a Nuu-chah-nulth origin site in Barkley Sound. Archaeology Press, Simon Fraser University, Burnaby

[CR29] McMillan AD, St. Claire DE (2012) Huu7̲ii: household archaeology at a Nuu-chah-nulth Village site in Barkley Sound. Archaeology Press, Simon Fraser University, Burnaby

[CR30] Menzies CR (2006). Traditional ecological knowledge and natural resource management.

[CR31] Miszaniec JI (2021) Assessing past ecological tolerance of Pacific salmon (*Oncorhynchus* spp.) and saffron cod (*Eleginus gracilis*) in northwest Alaska using vertebra width and length reconstructions. Archaeol. Anthropol. Sci. 13(6):1–14

[CR32] Monks GG, Monks GG (2017). Evidence of changing climate and subsistence strategies among the Nuu-chah-nulth of Canada’s West Coast. Climate Change and Human Responses: A Zooarchaeological Perspective.

[CR33] Moss ML (2012). Understanding variability in Northwest Coast faunal assemblages: beyond economic intensification and cultural complexity. J Isl Coast Archaeol.

[CR34] Moss ML, Cannon A (2011). The Archaeology of North Pacific Fisheries.

[CR35] Moss ML, Judd KG, Kemp BM (2014) Can salmonids (*Oncorhynchus *spp.) be identified to species using vertebral morphometrics? A test using ancient DNA from Coffman Cove, Alaska. J Archaeol Sci 41:879–889

[CR36] Ng JP, Cheung WWL (2022). Signature of climate-induced changes in seafood species served in restaurants. Environ Biol Fishes.

[CR37] Nims R, Butler VL (2019). The sablefish (*Anoplopoma fimbria*) of Čḯxwicən: socioenvironmental lessons from an unusually abundant species. J Archaeol Sci Rep.

[CR38] NPAFC (2021) NPAFC Pacific salmonid catch statistics (updated September 2021). North Pacific Anadromous Fish Commission, Vancouver

[CR39] Orchard TJ (2003). An application of the linear regression technique for determining length and weight of six fish taxa: the role of selected fish species in Aleut paleodiet.

[CR40] Orchard TJ, Szpak P, Moss ML, Cannon A (2011). Identification of salmon species from archaeological remains on the Northwest Coast. The Archaeology of North Pacific Fisheries.

[CR41] Pinsky ML, Worm B, Fogarty MJ, Sarmiento JL, Levin SA (2013). Marine taxa track local climate velocities. Science.

[CR42] Praetorius SK, Mix AC, Walczak MH, Wolhowe MD, Addison JA, Prahl FG (2015) North Pacific deglacial hypoxic events linked to abrupt ocean warming. Nature 527(7578):362–366. 10.1038/nature15753 10.1038/nature15753 26581293

[CR43] Rodrigues AT, McKechnie I, Yang DY (2018). Ancient DNA analysis of Indigenous rockfish use on the Pacific Coast: implications for marine conservation areas and fisheries management. PLoS ONE.

[CR44] Salmen-Hartley JU (2018) Towards a historical ecology of halibut fishing on the Northwest Coast. MA Thesis, University of Victoria, Victoria

[CR45] Sanchez GM (2020). Indigenous stewardship of marine and estuarine fisheries?: reconstructing the ancient size of Pacific herring through linear regression models. J Archaeol Sci Rep.

[CR46] Steneck RS, Pauly D (2019). Fishing through the Anthropocene. Curr Biol.

[CR47] Vigneron M (2021) Estimating Pile Perch (*Rhacochilus vacca*) size at Kakmakimilh (306T): potential evidence for stone walled tidal fish trap use by inhabitants of Kakmakimilh. Report submitted to the Kakmakimilh Archaeological Project, University of Victoria, Victoria

[CR48] Weatherdon LV, Ota Y, Jones MC, Close DA, Cheung WWL (2016). Projected scenarios for coastal First Nations’ fisheries catch potential under climate change: management challenges and opportunities. PLoS ONE.

[CR49] Wilson TJB, Cooley SR, Tai TC, Cheung WWL, Tyedmers PH (2020). Potential socioeconomic impacts from ocean acidification and climate change effects on Atlantic Canadian fisheries. PLoS ONE.

[CR50] Wright CA, Dallimore A, Thomson RE, Patterson RT, Ware DM (2005). Late Holocene paleofish populations in Effingham Inlet, British Columbia. Canada Palaeogeogr Palaeoclimatol Palaeoecol.

[CR51] Yanai RD, Battles JJ, Richardson AD, Blodgett CA, Wood DM, Rastetter EB (2010). Estimating uncertainty in ecosystem budget calculations. Ecosystems.

