# Supplementary material for: A palaeothermometer of ancient Indigenous fisheries reveals increases in mean temperature of the catch over five millennia
Source: Environ Biol Fishes. 2022 May 19;105(10):1381–97. doi: 10.1007/s10641-022-01243-7 (PMC9592643; doi:10.1007/s10641-022-01243-7)
Supplement: Supplementary file 2 — Supplementary file2 (DOCX 49 KB) [file 10641_2022_1243_MOESM2_ESM.docx]

**Supplementary Information for:**

**A Palaeothermometer of Ancient Indigenous Fisheries Reveals Increases in Mean Temperature of the Catch Over Five Millennia**

Dylan Hillis, Robert Gustas, Daniel Pauly, William W.L. Cheung, Anne K. Salomon & Iain McKechnie

**Supplementary Information: Tables**

**Table S1:** Fine screen (3.2 and 2 mm) column sample vertebrate data used in this study are from two archaeological sites, DfSi-16 (Ts’ishaa) and DfSh-7 (Huu7ii), as described in McKechnie (2005; 2012) and with archeological context further reported in McMillan and St. Claire (2005; 2012). Included in this table are site name, column sample name, approximate age range of each column sample, individual levels examined for faunal analysis, the total volume (L) of sediment recovered, and the total Number of Identified Specimens (NISP) per column sample. Note, the NISP for DfSi-16 and for DfSh-7 present data for fish only (i.e., excluding mammal, bird and unidentified specimens).

| Site Name | Column Sample | Approx. Age (cal yr BP) | No. of Examined Levels | Total Volume (litres) | NISP |
| --- | --- | --- | --- | --- | --- |
| DfSi-16 (Ts’ishaa) | N2-4/W102-104 | 1,500-250 | 10 | 40 | 2,857 |
| DfSi-16 (Ts’ishaa) | S14-16/W25-27 | 1,800-250 | 12 | 75 | 1,928 |
| DfSi-16 (Ts’ishaa) | S5-7/W11-13 | 1,000-250 | 12 | 24 | 941 |
| DfSi-16 (Ts’ishaa) | S56-57/W50-52 | 5,000-3,000 | 13 | 13 | 558 |
| DfSi-16 (Ts’ishaa) | S62-64/W62-64 | 5,000-3,000 | 5 | 20 | 695 |
| DfSh-7 (Huu7ii) | N10-12/E2-4 | 1,500-400 | 19 | 19 | 2,928 |
| DfSh-7 (Huu7ii) | N12-14/E6-8 | 1,500-400 | 12 | 12 | 2,406 |
| DfSh-7 (Huu7ii) | N18-20/E2-4 | 1,500-400 | 21 | 21 | 4,510 |
| DfSh-7 (Huu7ii) | N18-20/E6-8 | 1,500-400 | 21 | 21 | 8,507 |
| DfSh-7 (Huu7ii) | N10-12/E4-6 | 1,500-400 | 1 | 1 | 99 |
| DfSh-7 (Huu7ii) | N14-16/E15.5-16 | 1,500-400 | 6 | 6 | 1,730 |
| DfSh-7 (Huu7ii) | N18-20/E15.5-16 | 1,500-400 | 8 | 8 | 1,551 |
| DfSh-7 (Huu7ii) | N18-20/E26-28 | 1,500-400 | 9 | 9 | 1,790 |
| DfSh-7 (Huu7ii) | N18-20/E34-36 | 1,500-400 | 25 | 25 | 6,980 |
| DfSh-7 (Huu7ii) | N2-4/W18-20 | 5,000-3,000 | 25 | 25 | 11,439 |
| DfSh-7 (Huu7ii) | N4-6/E0-2 | 4,700-3,000 | 17 | 34 | 5,920 |

**Table S2:** Selection hierarchy for estimating the body mass of each species of fish present in the Barkley Sound zooarchaeological record.

| Rank | Source Type | Sources |
| --- | --- | --- |
| 1 | Measured archaeological specimens with body mass estimated from length-to-weight regression formulae | (McKechnie 2007a; McKechnie 2007b; Nims and Butler 2019; Orchard 2003; Salmen-Hartley 2018; Sanchez 2020) |
| 2 | Regionally specific fisheries independent scientific survey data | (Anderson et al. 2019) |
| 3 | Recreational length & weight data for dockside landings in Barkley Sound (Areas 23 & 123) | (DFO 2001) |
| 4 | Size and body mass data for specimens in the University of Victoria Zooarchaeology Comparative Collection | (McKenzie 2021) |
| 5 | Other archaeological and scientific literature | (Froese and Pauly 2021; NPAFC 2021) |

**Table S3:** Radiocarbon results for archaeological deposits associated with column sample data used in this study. Calibrated age-ranges are in calendar years before present (BP) at 2-sigma probability (95.4%) and were calibrated with Oxcal (Ramsey 2009) using the Intcal20 curve (Reimer et al. 2020).

| Site No. | Sample No. | Material | 14C Age (BP) | Calibrated Age-Range | Source |
| --- | --- | --- | --- | --- | --- |
| DfSi-16 | Beta-134655 | Charcoal | 1490±60 | 1517-1296 | 1 |
| DfSi-16 | Beta-134656 | Charcoal | 1800±60 | 1830-1545 | 1 |
| DfSi-16 | Beta-147071 | Charcoal | 3580±80 | 4141-3645 | 1 |
| DfSi-16 | Beta-147072 | Charcoal | 500±60 | 648-335 | 1 |
| DfSi-16 | Beta-147073 | Charcoal | 5050±60 | 5916-5608 | 1 |
| DfSi-16 | Beta-147074 | Charcoal | 1230±90 | 1295-961 | 1 |
| DfSi-16 | Beta-147075 | Charcoal | 690±60 | 723-552 | 1 |
| DfSi-16 | Beta-158739 | Charcoal | 430±60 | 545-315 | 1 |
| DfSi-16 | Beta-158740 | Charcoal | 3000±70 | 3365-2968 | 1 |
| DfSi-16 | Beta-158741 | Charcoal | 4470±70 | 5309-4877 | 1 |
| DfSi-16 | Beta-158742 | Charcoal | 3330±70 | 3817-3396 | 1 |
| DfSi-16 | Beta-158743 | Charcoal | 4430±80 | 5294-4861 | 1 |
| DfSi-16 | Beta-158744 | Charcoal | 3050±70 | 3442-3009 | 1 |
| DfSi-16 | Beta-158745 | Charcoal | 4080±70 | 4822-4420 | 1 |
| DfSi-16 | Beta-158746 | Charcoal | 1620±60 | 1692-1374 | 1 |
| DfSi-16 | Beta-158747 | Charcoal | 4160±70 | 4849-4450 | 1 |
| DfSh-7 | Beta-195633 | Charcoal | 640±50 | 670-548 | 2 |
| DfSh-7 | Beta-195634 | Charcoal | 740±70 | 788-554 | 2 |
| DfSh-7 | Beta-195635 | Charcoal | 470±60 | 630-320 | 2 |
| DfSh-7 | Beta-195636 | Charcoal | 820±60 | 905-662 | 2 |
| DfSh-7 | Beta-195637 | Charcoal | 3190±60 | 3561-3250 | 2 |
| DfSh-7 | Beta-195638 | Charcoal | 1170±70 | 1271-936 | 2 |
| DfSh-7 | Beta-195639 | Charcoal | 1330±50 | 1344-1128 | 2 |
| DfSh-7 | Beta-195640 | Charcoal | 1560±60 | 1546-1311 | 2 |
| DfSh-7 | Beta-195641 | Charcoal | 4280±70 | 5046-4580 | 2 |
| DfSh-7 | Beta-195642 | Charcoal | 1230±60 | 1288-994 | 2 |
| DfSh-7 | Beta-221950 | Charcoal | 610±40 | 657-541 | 2 |
| DfSh-7 | Beta-221951 | Charcoal | 410±70 | 540-310 | 2 |
| DfSh-7 | Beta-221952 | Charcoal | 370±70 | 523-297 | 2 |
| DfSh-7 | Beta-221953 | Charcoal | 2830±60 | 3145-2781 | 2 |
| DfSh-7 | Beta-221954 | Charcoal | 1190±60 | 1269-960 | 2 |
| DfSh-7 | Beta-221955 | Charcoal | 710±40 | 722-561 | 2 |
| DfSh-7 | Beta-221956 | Charcoal | 1290±70 | 1342-1059 | 2 |
| DfSh-7 | Beta-221957 | Charcoal | 670±70 | 723-540 | 2 |
| DfSh-7 | Beta-221959 | Charcoal | 990±50 | 1046-752 | 2 |
| DfSh-7 | Beta-221960 | Charcoal | 3690±70 | 4236-3840 | 2 |
| DfSh-7 | Beta-221961 | Charcoal | 710±60 | 731-554 | 2 |
| DfSh-7 | Beta-221962 | Charcoal | 3810±50 | 4406-4014 | 2 |
| DfSh-7 | Beta-221963 | Charcoal | 3810±80 | 4419-3978 | 2 |
| DfSh-7 | Beta-236288 | Charcoal | 1060±40 | 1064-911 | 2 |
| DfSh-7 | Beta-236289 | Charcoal | 920±50 | 923-731 | 2 |
| DfSh-7 | CAMS-28075 | Charcoal | 2260±50 | 2352-2146 | 2 |
| DfSh-7 | CAMS-97176 | Charcoal | 3585±40 | 4060-3724 | 2 |
| DfSh-7 | CAMS-97177 | Charcoal | 3735±35 | 4231-3977 | 2 |
| DfSh-7 | CAMS-97181 | Charcoal | 4210±35 | 4851-4620 | 2 |
| DfSh-7 | CAMS-97182 | Charcoal | 4415±35 | 5275-4865 | 2 |
| DfSh-7 | CAMS-97186 | Charcoal | 3100±35 | 3390-3215 | 2 |
| DfSh-7 | CAMS-97191 | Charcoal | 350±45 | 495-310 | 2 |
| DfSh-7 | CAMS-97197 | Charcoal | 1310±35 | 1296-1176 | 2 |
| DfSh-7 | CAMS-97198 | Charcoal | 1230±35 | 1270-1065 | 2 |
| DfSh-7 | CAMS-97203 | Charcoal | 1385±35 | 1354-1179 | 2 |
| DfSh-7 | CAMS-97204 | Charcoal | 1300±35 | 1296-1154 | 2 |
| Note: source 1 refers to original radiocarbon data for DfSi-16 presented in McMillan and St. Claire (2005), while source 2 refers to radiocarbon data for DfSh-7 as presented in McMillan and St. Claire (2012). | | | | | |

**Table S4:** Comparison of the relative proportion of the Minimum Number of Individuals (% MNI) and the relative proportion of the catch (% Biomass) for each archaeological site and temporal period under consideration. % Biomass is calculated using the 25th quartile body mass estimate multiplied by MNI counts for each taxonomic grouping.

|  | Ts’ishaa  % MNI | | Ts’ishaa  % Biomass | | Huu7ii  % MNI | | Huu7ii  % Biomass | |
| --- | --- | --- | --- | --- | --- | --- | --- | --- |
| Approx. Age (cal yr BP) | 5,000-3,000 | 1,800-250 | 5,000-3,000 | 1,800-250 | 5,000-3,000 | 1,500-400 | 5,000-3,000 | 1,500-400 |
| Anchovy | 7 | 16 | <1 | 1 | 7 | 13 | <1 | <1 |
| Dogfish | 3 | 4 | 4 | 6 | 3 | 6 | 9 | 9 |
| Flatfish | 2 | 2 | 1 | 1 | <1 | 3 | <1 | 2 |
| Greenling | 24 | 13 | 7 | 4 | 11 | 12 | 5 | 3 |
| Hake | <1 | 3 | <1 | <1 | <1 | 6 | <1 | 1 |
| Halibut | 4 | 2 | 25 | 15 | <1 | <1 | 3 | 2 |
| Herring | 27 | 23 | 4 | 4 | 62 | 28 | 15 | 4 |
| Lingcod | 5 | 4 | 9 | 7 | <1 | 2 | 1 | 3 |
| Perch | 10 | 7 | 7 | 5 | 5 | 3 | 5 | 2 |
| Ratfish | 1 | 1 | <1 | 1 | 1 | 1 | 1 | 1 |
| Rockfish | 11 | 13 | 5 | 5 | 4 | 10 | 3 | 4 |
| Sablefish | 1 | 2 | 1 | 1 | <1 | 3 | <1 | 2 |
| Salmon | 6 | 7 | 36 | 41 | 6 | 10 | 57 | 60 |
| Sculpin | <1 | 4 | <1 | 10 | 1 | 3 | 2 | 7 |

**Table S5:** Comparison of the relative proportion of the Minimum Number of Individuals (% MNI) and the relative proportion of the catch (% Biomass) for each archaeological site and temporal period under consideration. % Biomass is calculated using the 75th quartile body mass estimate multiplied by MNI counts for each taxonomic grouping.

|  | Ts’ishaa  % MNI | | Ts’ishaa  % Biomass | | Huu7ii  % MNI | | Huu7ii  % Biomass | |
| --- | --- | --- | --- | --- | --- | --- | --- | --- |
| Approx. Age (cal yr BP) | 5,000-3,000 | 1,800-250 | 5,000-3,000 | 1,800-250 | 5,000-3,000 | 1,500-400 | 5,000-3,000 | 1,500-400 |
| Anchovy | 7 | 16 | <1 | <1 | 7 | 13 | <1 | <1 |
| Dogfish | 3 | 4 | 3 | 6 | 3 | 6 | 9 | 9 |
| Flatfish | 2 | 2 | 1 | 2 | <1 | 3 | <1 | 3 |
| Greenling | 24 | 13 | 9 | 5 | 11 | 12 | 9 | 5 |
| Hake | <1 | 3 | <1 | 2 | <1 | 6 | <1 | 5 |
| Halibut | 4 | 2 | 32 | 21 | <1 | <1 | 4 | 3 |
| Herring | 27 | 23 | 3 | 3 | 62 | 28 | 14 | 3 |
| Lingcod | 5 | 4 | 13 | 11 | <1 | 2 | 1 | 5 |
| Perch | 10 | 7 | 5 | 4 | 5 | 3 | 5 | 2 |
| Ratfish | 1 | 1 | <1 | 1 | 1 | 1 | 1 | 1 |
| Rockfish | 11 | 13 | 7 | 9 | 4 | 10 | 6 | 8 |
| Sablefish | 1 | 2 | <1 | 1 | <1 | 3 | <1 | 1 |
| Salmon | 6 | 7 | 23 | 30 | 6 | 10 | 49 | 48 |
| Sculpin | <1 | 4 | <1 | 6 | 1 | 3 | 2 | 5 |

**Supplementary Information: References**

Anderson SC, Keppel EA, Edwards AM (2019) A reproducible data synopsis for over 100 species of British Columbia groundfish. Fisheries and Oceans Canada, Nanaimo.

DFO (2001) West Coast Vancouver Island sport fishery creel survey statistics 2001 and historical data 1984-2000 / by DM Lewis. Fisheries and Oceans Canada, Pacific Biological Station, Nanaimo.

Froese R, Pauly D (2021) FishBase. In https://fishbase.mnhn.fr/search.php

McKechnie I (2005) Column Sampling and the Archaeology of Small Fish at Ts’ishaa. In: AD McMillan & D St. Claire (eds.), Ts'ishaa: Archaeology and Ethnography of a Nuu-chah-nulth Origin Site in Barkley Sound pp. 206–223. Archaeology Press, Simon Fraser University, Burnaby.

McKechnie I (2007a) Investigating the complexities of sustainable fishing at a prehistoric village on western Vancouver Island, British Columbia, Canada. J. Nat. Conserv. 15(3):208-222.

McKechnie I (2007b) Vertebrate faunal analysis at Himayis (205T, DeSi-17) and the Clarke Island Defensive Site (212T, DeSi-26), Broken Group Islands, Pacific Rim National Park Reserve. Parks Canada, Cultural Resource Services, Victoria, pp 1-68.

McKechnie I (2012) Zooarchaeological Analysis of the Indigenous Fishery at the Huu7ii Big House and Back Terrace, Huu-ay-aht Territory, Southwestern Vancouver Island. In: AD McMillan & D St. Claire (eds.), Huu7ii: Household Archaeology at a Nuu-chah-nulth Village Site in Barkley Sound. pp. 154–186. Archaeology Press, Simon Fraser University, Burnaby.

McKenzie K (2021) University of Victoria - Zooarchaeology Lab Collection v1.1. University of Victoria, Victoria, <https://doi.org/10.5886/jej09d>

McMillan AD, St. Claire DE (2005) Ts’ishaa: archaeology and ethnography of a Nuu-chah-nulth origin site in Barkley Sound. Archaeology Press, Simon Fraser University, Burnaby.

McMillan AD, St. Claire DE (2012) Huu7ii: household archaeology at a Nuu-chah-nulth Village site in Barkley Sound. Archaeology Press, Simon Fraser University, Burnaby.

Nims R, Butler VL (2019) The sablefish (*Anoplopoma fimbria*) of Čḯxwicən: Socioenvironmental lessons from an unusually abundant species. J. Archaeol. Sci. Rep. 23:1187-1196 <https://doi.org/10.1016/j.jasrep.2018.06.028>

NPAFC (2021) NPAFC Pacific salmonid catch statistics (updated September 2021). North Pacific Anadromous Fish Commission, Vancouver.

Orchard TJ (2003) An application of the linear regression technique for determining length and weight of six fish taxa: The role of selected fish species in Aleut paleodiet. Vol 1172. British Archaeological Reports, Oxford.

Ramsey BC (2009) Bayesian Analysis of Radiocarbon Dates. Radiocarbon 51(1):337-360  [https://doi.org/10.1017/S0033822200033865](%09https://doi.org/10.1017/S0033822200033865)

Reimer PJ, et al. (2020) The IntCal20 Northern Hemisphere Radiocarbon Age Calibration Curve (0–55 cal kBP). Radiocarbon 62(4):725-757 <https://doi.org/10.1017/RDC.2020.41>

Salmen-Hartley JU (2018) Towards a historical ecology of halibut fishing on the Northwest Coast. MA Thesis, Department of Anthropology, University of Victoria, Victoria. http://hdl.handle.net/1828/10034

Sanchez GM (2020) Indigenous stewardship of marine and estuarine fisheries?: Reconstructing the ancient size of Pacific herring through linear regression models. J. Archaeol. Sci. Rep. 29:102061 <https://doi.org/10.1016/j.jasrep.2019.102061>
